# Quality Improvement in Surgery Combining Lean Improvement Methods with Teamwork Training: A Controlled Before-After Study

**DOI:** 10.1371/journal.pone.0138490

**Published:** 2015-09-18

**Authors:** Eleanor Robertson, Lauren Morgan, Steve New, Sharon Pickering, Mohammed Hadi, Gary Collins, Oliver Rivero Arias, Damian Griffin, Peter McCulloch

**Affiliations:** 1 Nuffield Department of Surgical Sciences, University of Oxford, Oxford, United Kingdom; 2 Saïd Business School, University of Oxford, Oxford, United Kingdom; 3 Warwick Medical School, University of Coventry and Warwick, Warwick, United Kingdom; 4 Centre for Statistics in Medicine, University of Oxford, Oxford, United Kingdom; 5 Nuffield Department of Population Health, University of Oxford, Oxford, United Kingdom; 6 Red de Investigación de Servicios Sanitarios en Cronicidad (REDISSEC), Madrid, Spain; Universita' degli Studi di Napoli Federico II, ITALY

## Abstract

**Background:**

To investigate the effectiveness of combining teamwork training and lean process improvement, two distinct approaches to improving surgical safety. We conducted a controlled interrupted time series study in a specialist UK Orthopaedic hospital incorporating a plastic surgery team (which received the intervention) and an Orthopaedic theatre team acting as a control.

**Study Design:**

We used a 3 month intervention with 3 months data collection period before and after it. A combined teamwork training and lean process improvement intervention was delivered by an experienced specialist team. Before and after the intervention we evaluated team non-technical skills using NOTECHS II, technical performance using the glitch rate and WHO checklist compliance using a simple 3 point scale. We recorded complication rate, readmission rate and length of hospital stay data for 6 months before and after the intervention.

**Results:**

In the active group, but not the control group, full compliance with WHO Time Out (T/O) increased from 14 to 71% (p = 0.032), Sign Out attempt rate (S/O) increased from 0% to 50% (p<0.001) and Oxford NOTECHS II scores increased after the intervention (P = 0.058). Glitch rate decreased in the active group and increased in the control group (p = 0.001). Complications and length of stay appeared to rise in the control group and fall in the active group.

**Conclusions:**

Combining teamwork training and systems improvement enhanced both technical and non-technical operating team process measures, and were associated with a trend to better safety outcome measures in a controlled study comparison. We suggest that approaches which address both system and culture dimensions of safety may prove valuable in reducing risks to patients.

## Introduction

The provision of safe, reliable healthcare has become a national and international priority for both the developed[1, 2] and the developing world[3]. It is now more than 20 years since the widespread recognition that the delivery of healthcare in complex systems which have not been carefully and rationally designed to minimise risk can and frequently does result in harm[4], with levels of risk equivalent to micro light aircraft flight[5]. Attempts have been made to improve the sustainable delivery of safe healthcare using a variety of approaches. It has been recognised that surgery is one of the most challenging areas of healthcare in which to provide safe and reliable care, with incident analysis studies consistently finding a higher level of harm than in medical specialities[6–8].

The concept of resilience demands consistent, safe delivery of output despite changing circumstances[9, 10]. Studies of high reliability organisations (HROs) have highlighted both the care with which processes are designed, defined and standardised, and the emphasis put on optimising human teamwork and communications. There have been attempts to translate concepts from HROs (aviation, nuclear, manufacturing and petrochemical) into the healthcare industry through improvement interventions of various types. Crew resource management (CRM) courses to improve teamwork and communications have been translated to healthcare from aviation, and aim to improve human reliability in stressful environments through communication strategies and behavioural models which improve the culture of the working environment. The provision of this kind of teamwork training in the operating theatre environment has become increasingly common in healthcare. A recent systematic literature review [11] found that such training clearly improved teamwork and technical performance, but evidence of clinical benefit was less clear cut. Another stream of safety improvement work has emphasised the importance of reducing system-derived error, and has relied on lessons particularly from “lean” manufacturing industry approaches such as the Toyota Production System (TPS) [12–14], where improved efficiency is achieved by allocating more responsibility to the frontline staff. This approach has been championed in the US by organisations such as IHI, and in the British NHS through ‘the productive ward’ and ‘the productive operating theatre’ series [15, 16]. Both teamwork and “lean” approaches can have positive effects, but little is known about the effects when they are delivered together. The hypothesis that safety interventions based on enhancing team culture might synergise with those based on rationalising systems has been proposed as a prediction of the “3D” model, which recognises three “dimensions” of safety, namely culture, system and technology[17]. To test this hypothesis, we conducted a multi-site programme of 5 linked prospective controlled studies evaluating different team and system interventions and their combinations (the Safer Delivery of Surgical Services or S3 study). Two studies (of which this was one) used combinations of teamwork training with a systems improvement intervention, whilst three studies examined single intervention approaches (either teamwork training, “lean” or a systems improvement approach based on redesign led by professional ergonomists). Each study evaluated both process improvement (including the quality of the WHO surgical safety checklist process[18–20]) and clinical outcome improvement. In this study we examine the effect of an intervention which combines teamwork training with “lean” process improvement in improving safety and reliability in operating theatre teams.

## Methods

### Setting

The study was conducted in a specialist elective orthopaedic and reconstructive surgery hospital, with 106 beds and six operating theatres. There was no overlap of senior staff members between the active and control groups, and care was taken to avoid “contamination” between the teams as far as compatible with normal social contact. The active team specialised in plastic and reconstructive surgery (elective upper limb surgery, osteomyelitis and sarcoma surgery), and the control team performed lower limb elective orthopaedic surgery (primary and revision hip and knee arthroplasty and arthroscopic knee surgery). The seniority and skill-mix of the nursing, surgical and anaesthetic staff were similar in active and control groups, and the facilities for pre and post-operative care and patient recovery after anaesthesia were identical.

### Study Design

This was a controlled before-after study, with 6 months clinical and 3 months observational process data collection either side of a 3 month intervention phase (active only).The study ran from 04.2011 to 07.2012, with clinical data collection periods from 4/11-9/11 and 1/12 to 7/12, and observational data collection periods from 7/11 to 9/11 and 1/12 to 3/12.

### Intervention

The Safer Delivery of Surgical Services (S3) Programme pre-assigned each study site with a style of intervention, either single or combined. Two of five studies in the Programme studied combination interventions, and the other three single intervention approaches. The choice of intervention was made prior to baseline data collection. In this study the intervention tested was lean process engineering in combination with teamwork training based on aviation style crew resource management.

### Lean process engineering

A senior management consultant (SN) delivered one half day training session to core members of the frontline healthcare team(11 staff attended). Following this, frequent on-site coaching and support was provided to frontline staff project groups by study team members over the course of the intervention period. The intervention explained the nature of lean process engineering and its’ relevance to healthcare; it covered the main lean concepts and operational techniques including Muda, Poka-Yoke, Genchi Genbutsu, Kaizen, flow, Just in time, respect and teamwork, process mapping, PDCA cycles and a philosophy of continuous improvement. Frontline staff were encouraged to pin-point areas of focus for improvement work.

### Teamwork training

The teamwork training was delivered by external consultants, in one morning and two evening sessions (20 staff attended one session each). The training consisted of educational content on the aetiology of human error from a psychological perspective, together with a discussion of teamwork based on the aviation CRM model, including the importance of sharing situational awareness, flat hierarchy, formal communications protocols and checklists. Following this, active theatres received 5 days in-theatre coaching spread over 6 weeks, and focusing on supporting the team in the optimum completion of the WHO surgical safety checklist process, and other non-technical skills.

### Outcome Measures

We assessed the effect of the intervention on work processes with 3 observational process measures: Non-technical skills team assessment (Oxford NOTECHS II) [18]; count of operative process glitches [19] and quality of WHO checklist completion. Clinical outcome was measured using Length of hospital stay, complication rate and re—admission rate within 90 days. A large convenience sample of operations was studied during the three months immediately before and after the intervention period. Each operation was observed in full by two observers; one with a clinical and one with a human factors (HF) background, who had all undergone two months of training to aid harmonisation of data collection. Intra-operative observation began when the patient entered theatre and ended when they left the operating theatre. Data collection booklets for each surgical procedure were developed [21] to record observational data. At the end of the operation, these results were entered in to a secure de-identified database. Observers were not blinded to study arm, as this was impractical within the constraints of the study.

### Oxford NOTECHS II

The operating team’s non-technical skills were assessed through the Oxford NOTECHS II behavioural rating scale as previously described]. Briefly, each of 3 sub-teams: (nursing, surgical and anaesthetic) is scored on a 1–8 scale against 4 behavioural parameters: Leadership & management; Teamwork & cooperation; Problem solving & decision making; and situational awareness[22]. This gives a theoretical maximum score of 96 for the entire team.

### Glitches

Glitches are defined as deviations from the recognised process with the potential to reduce quality or speed, including interruptions, omissions and changes, whether or not these actually affected the outcome of the procedure. The glitches were collected independently by each observer individually noting the time and detail of the glitch and assigning it to one of 13 previously described categories[3]. The detail of the glitch (e.g. ‘diathermy not plugged in when surgeon trying to use it’) along with the associated time point was recorded. A glitch rate per hour (total number of glitches/operation duration) was calculated for each operation, allowing operations of differing durations to be compared.

### WHO Surgical Safety Checklist

To evaluate the quality of WHO Surgical Safety Checklist performance the observers collected information as to whether the time-out (T/O) and sign-out (S/O) were attempted[20], i.e. whether an attempt was made to complete these parts of the checklist. In addition, the quality of the checklist performance was evaluated using three simple markers: whether all required items were completed, whether all team members were present and whether there was active team participation[20]. We report the proportion of observed operations in which all three markers were present.

### Clinical Outcome data

Hospital episode statistics data were extracted for all patients undergoing operations in the relevant operating theatres under the involved Consultants during the 6 months periods immediately before and after the intervention. This therefore represents a larger group of patients, of which those whose operations were observed represented a convenience sample. The following data for each patient were independently extracted by Trust clerical staff and supplied to the research team in anonymised form: age; sex; diagnosis; consultant; operation; operating time; length of hospital stay; complications (any) and nature; readmission within 90 days re-operation. Comparisons between active and control groups were made for length of stay and number (%) of patients with any complication and readmissions.

### Data analysis

Differences between the control and active arms for process data were assessed using two-way analysis of variance (Group × Time), with intervention (control versus active) and time (pre-intervention versus post-intervention) as factors. The differences in before/after change in the active and control groups were assessed using the Group x Time interaction. F values were obtained by dividing by the residual variance. Pre- and post-intervention differences between active and control groups are reported as 95% confidence intervals. All statistical analyses were carried out in R (version 3.0.1). For clinical outcome data, baseline demographic information was summarised using descriptive statistics. T-tests for mean age and chi-square test for gender distribution were used to compare the before and after periods. Binary clinical outcome variables in the before and after periods were compared using Odds ratios and 95% confidence intervals from a logistic regression, and mean length of stay using linear regression, both adjusted for age and gender. This statistical analysis was conducted in Stata version 12 [StataCorp (2011).

### Ethics

Patients whose operations were observed were informed of the possibility of observations taking place and given opportunity to opt out if they wished. Staff in the theatres undergoing observation were given information on the study and provided written consent before observations took place. The study was approved by Oxford A Ethics Committee (REC:09/H0604/39).

## Results

### Intervention choice

Following the training sessions, the frontline theatre staff were encouraged to develop their own agenda for change, and decided on a number of topic areas (Table 1). Not all of these projects were successfully completed, and the relevance of projects to patient safety outcomes varied, but active engagement of the staff in selecting the programme of work was considered an important part of the intervention.

**Table 1:** Projects arising from the intervention.

Project title	Project aim	Outcome
Briefing	Begin a pre-list briefing to understand what work is planned for the whole list	Institution of routine pre-list briefing
WHO-checklist use	Improve reliability of WHO time- and sign-out	Standardisation of process: when it should occur and who should be involved
Debriefing	Feeding information back in to the system to reduce repetition of error and glitches	Production of a de-briefing feedback reporting system
Equipment procurement	Reduce waste of stock drift and improve financial planning	Development of new intra-operative recording sheets to reduce loss of stock
Prep room organisation	Standardisation of layout of prep rooms to reduce waste	Standardisation of prep rooms
List design	Improve the amount of information available on the operating lists	Unable to change due to impending new computer system
Awareness of sterile services function	Reveal previously hidden processes involved in sterile services	Presentation and Q&A session with TSSU staff

Project groups analysed their problem, collected data to measure it and developed solutions with the assistance of the S3 team under the supervision of the experts who provided training. New systems of working underwent iterative PDCA (plan, do, check, act) cycles of rapid testing until apparent stability was reached.

### 1.1 Outcome measure results

#### Study characteristics

One hundred and fifty-one operations were performed in the active group before intervention and 136 afterwards, compared to 418 and 355 in the same periods in the control group. A total of 96 operations were observed, 51 (26 before and 25 after intervention) in the control arm and 45 (21 before and 24 after) in the active arm. The mean operating time was longer in the active arm than the control arm (overall mean 2 hr 17 mins vs 1 hr 36 mins) but there was no significant change in the operating time after intervention in either group.

#### Oxford NOTECHS II

**Fig 1:**
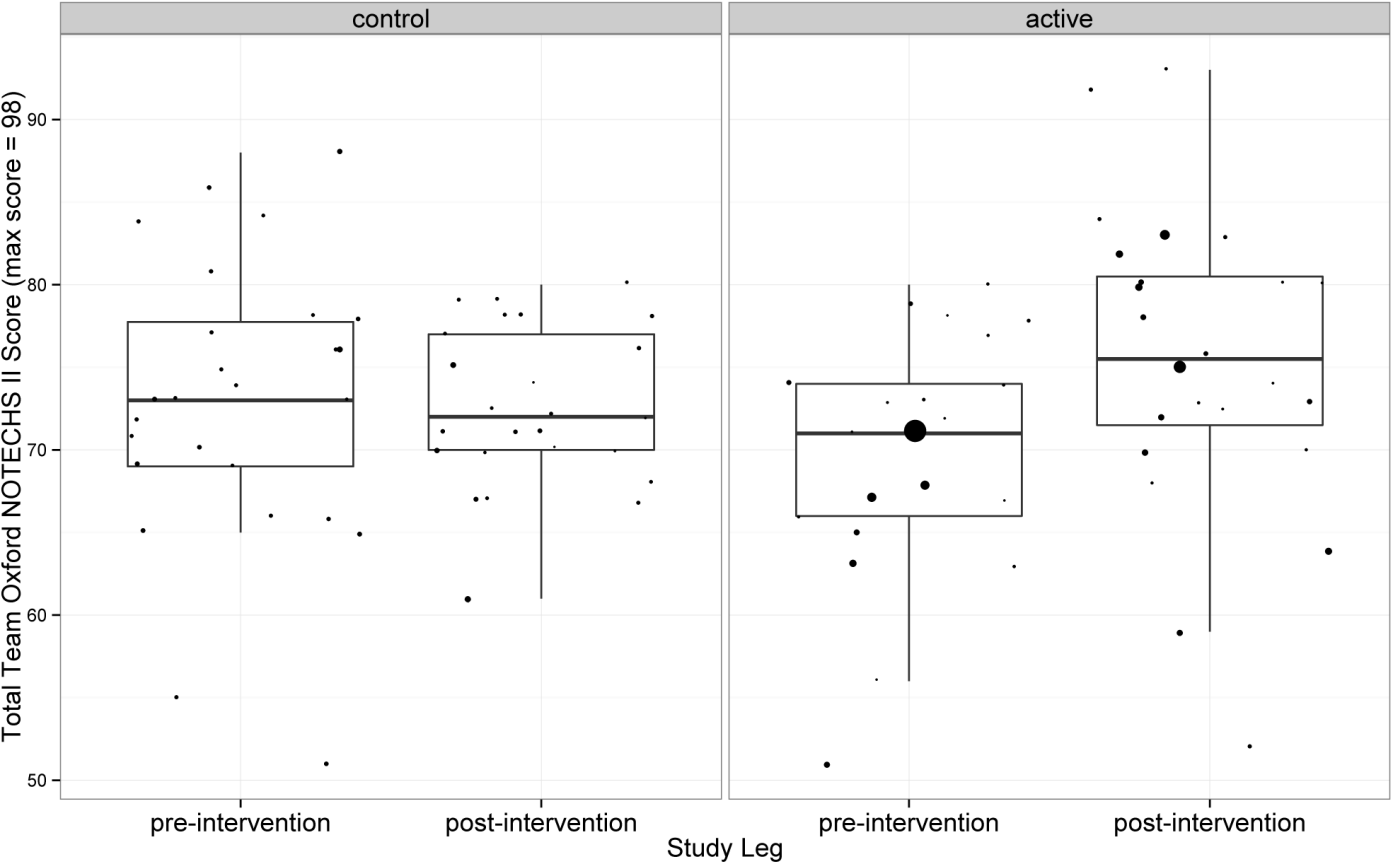
Team Oxford NOTECHS II scores.

Mean Oxford NOTECHS II score increased from 69.81 before to 75.56 after the intervention in the active group (difference = 5.75; 95% confidence interval 0.68 to 10.82) whilst it was static (72.88 before and 72.54 after) in the control group (difference = -0.36; 95% confidence interval -4.28 to 3.59) (Fig 1). The difference between the change in the active and control groups was of borderline significance (p = 0.058). Sub team analysis revealed that between-group differences in mean Oxford NOTECHS II scores were non-significant for surgeons (p = 0.101) and anaesthetists (p = 0.876) whilst statistically significant for nurses (difference 2.22; 95% CI 0.22 to 4.24; p = 0.016).

#### Glitch Rate

**Fig 2:**
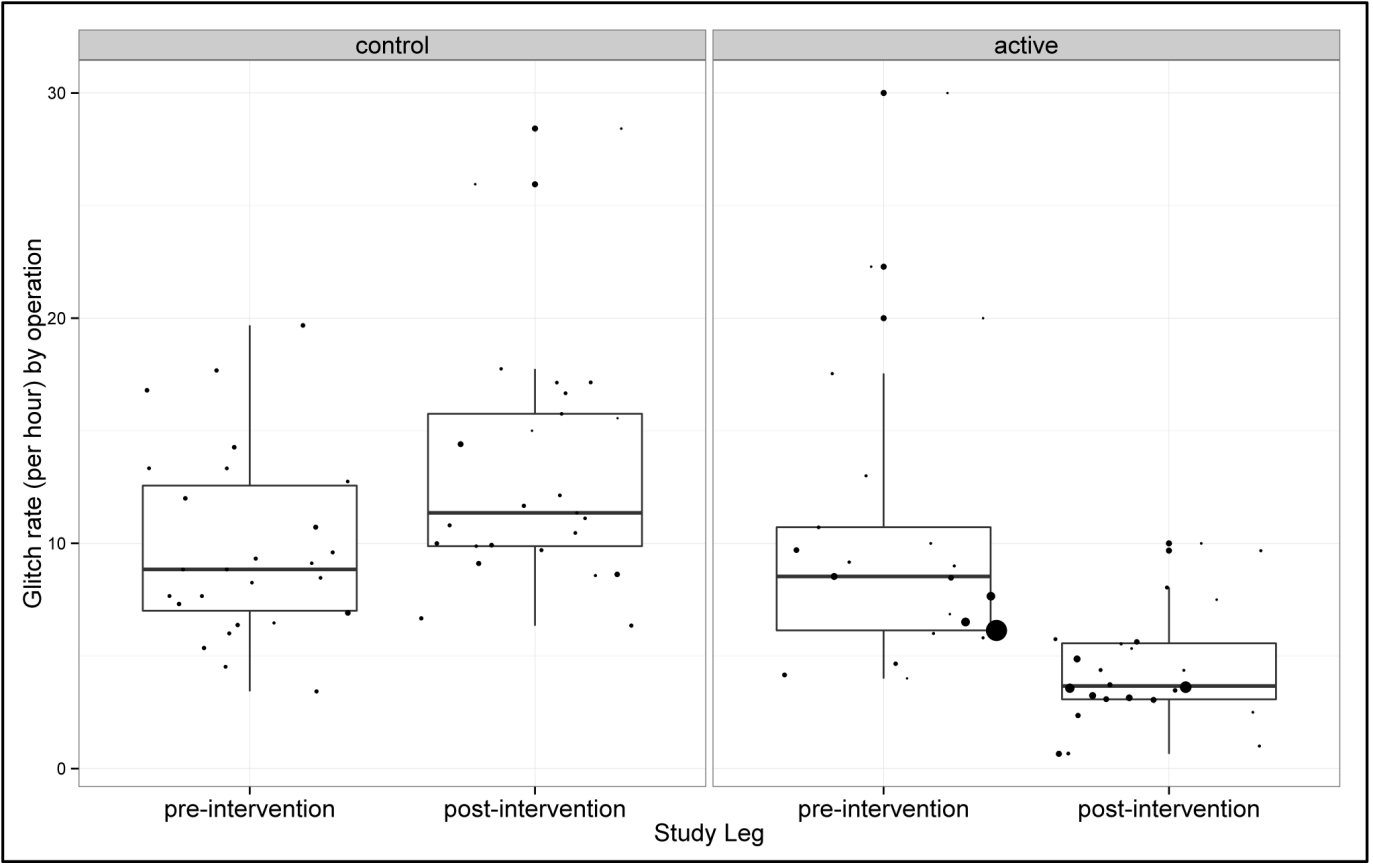
Glitch rate per hour.

**Fig 3:**
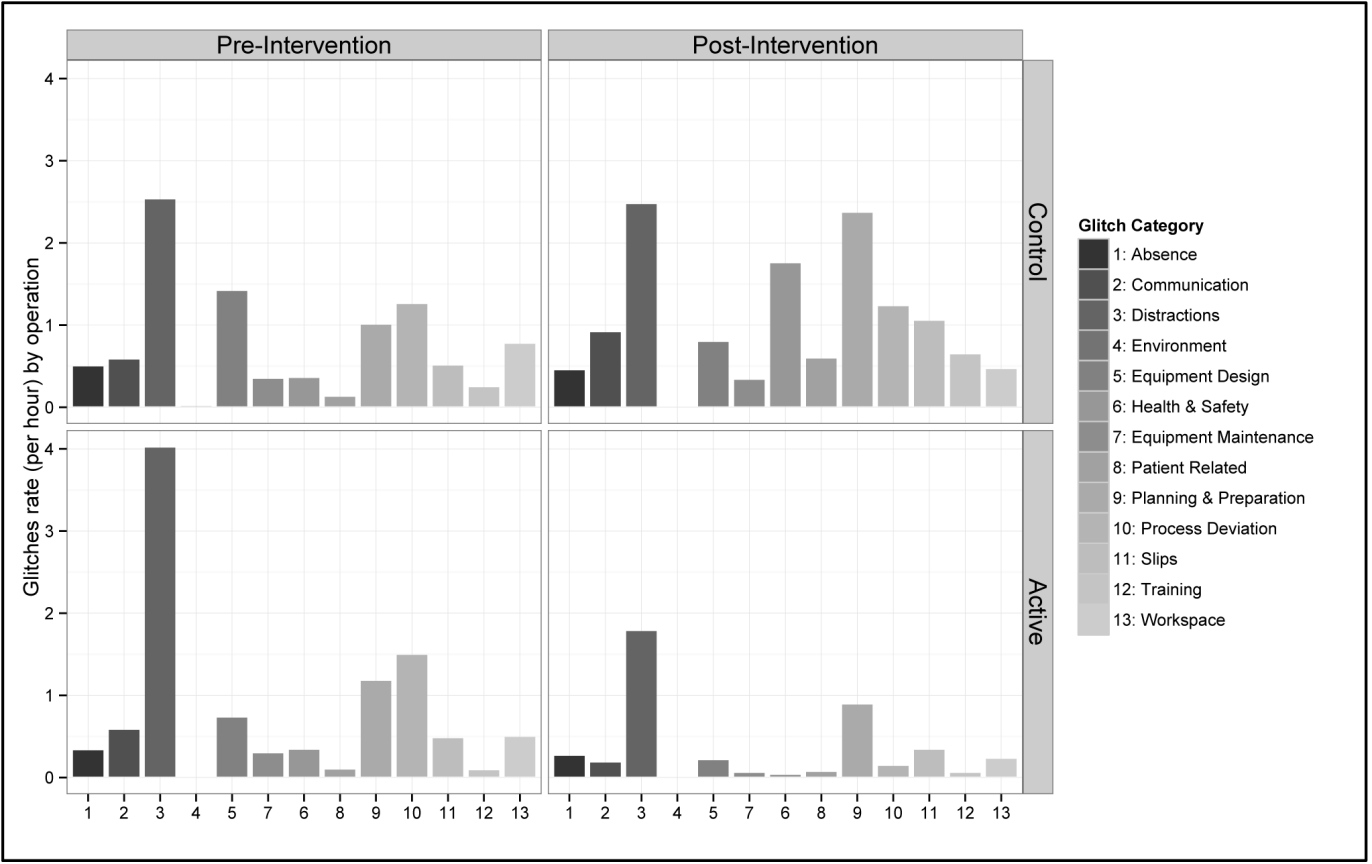
Mean incidence rate of different glitch categories.

The mean glitch rate was 10.48 (sd = 6.68) glitches per hour in the active group and 9.79 (sd = 4.12) glitches per hour in the control group before the intervention. After the intervention mean glitch rate decreased to 4.38 (sd = 2.50) glitches per hour in the active group (difference = -6.10; 95% CI -9.29 to -2.92) whilst in the control group it rose to 13.20 (sd = 5.37) glitches per hour (difference = 4.87; 95% CI 0.70 to 6.12). The difference between the two groups was statistically significant (p<0.001, Fig 2). We analysed the change in different categories of glitch in the two groups (Fig 3). The fall in Distractions after the intervention in the active group was very clear, whilst there was no change in these glitches in the control group.

#### WHO surgical safety checklist time out compliance and quality

**Fig 4:**
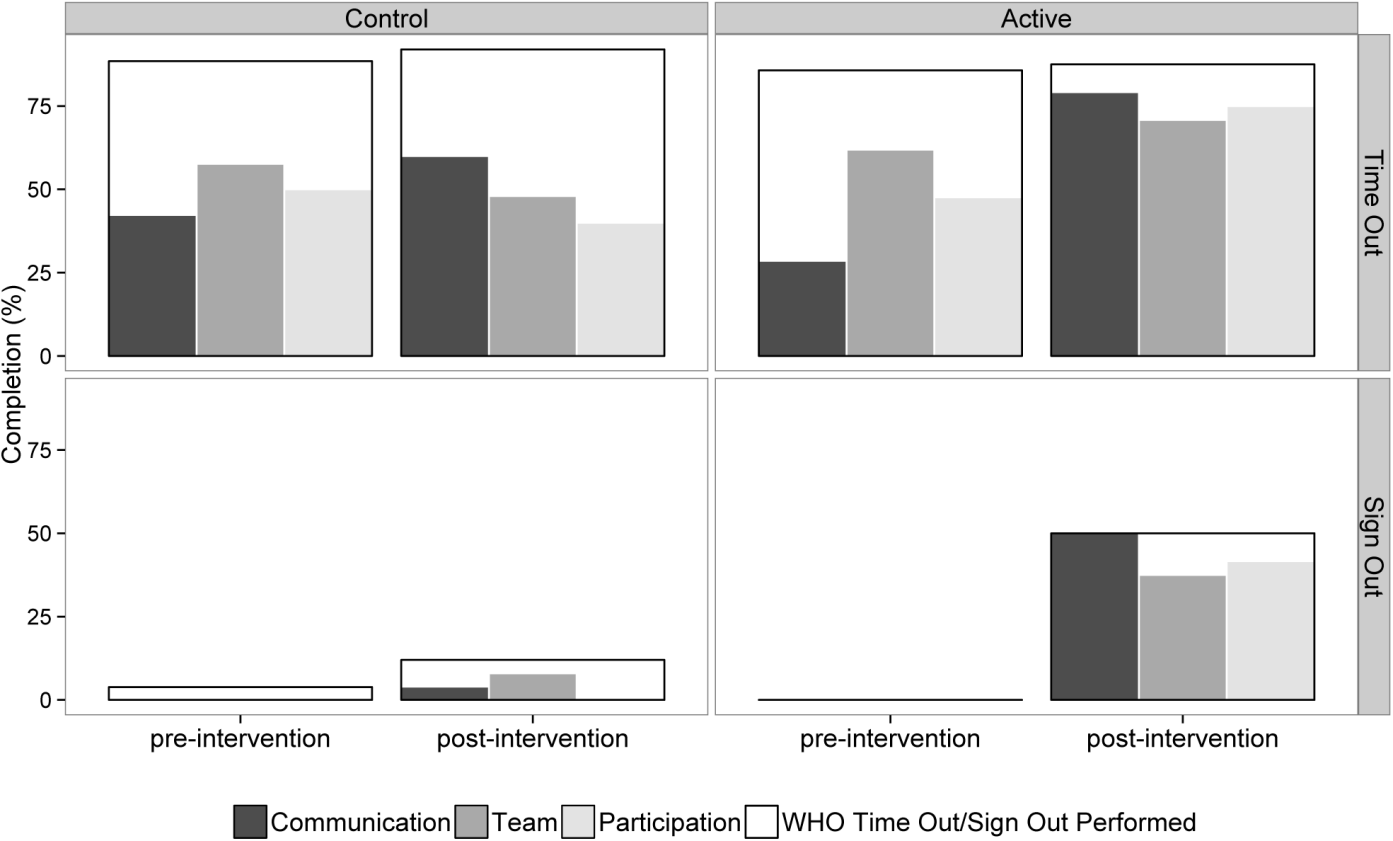
Frequency and quality of WHO time and sign-out checklist completion.

Of the 96 observed operations, WHO time-out (T/O) was attempted in 85(88%) and sign out (S/O) in 16(17%). There was no significant difference in the high attempt rate of T/O between pre (18/21; 86%) and post (21/24; 88%) intervention groups in either the active arm (difference = 2%; 95% CI -20% to 24%; p = 1), and pre (23/26; 88%) and post (23/25; 92%) or the control arm (difference = 4%; 95% CI -16% to 23%; p = 1). There was however a large change in the quality of the WHO time out compliance in the intervention group, with communication increasing from 29% to 79%, all team present increasing from 62% to 71% and active participation from 48% to 75% (Fig 4). All three components of T/O were completed in 3/21 (14%) cases in the pre-intervention active arm, which increased to 17/24 (71%) in the post-intervention phase (difference = 57%; 95% CI 29% to 85%). All three components of T/O were completed in 5/26 (19%) cases in the pre-intervention control arm, which increased to 7/25 (28%) in the post-intervention phase (difference = 9%; 95% CI -18% to 36%). The difference between the change in the active and control groups was significant (p = 0.032).

There was a significant difference in the attempt rate of S/O between pre (0/21; 0%) and post (12/24; 50%) in the active arm (difference = 50%; 95% CI 26% to 75%; p<0.001). There was no significant different between the pre (1/26; 4%) and post (3/25; 12%) in the control arm (difference = 8%; 95% CI -10% to 27%; p = 0.574). The difference between the change in the active and control groups was however not significant (p = 0.093).

#### Clinical Outcome Measures

Both complications and length of stay (LOS) fell after the intervention in the active group, whilst both rose in the control group (Table 2). The difference in before/after change between the two groups was however not significant. Both intervention and control groups experienced a rise in readmissions after the intervention, but there was no significant difference in the extent of this between the two groups (Table 2).

**Table 2:** Summary outcome measures. Data shown are mean (SD) unless otherwise specified.

	CONTROL		INTERVENTION		p value
	Pre- Int	Post-Int	Pre- Int	Post-Int	(Δ CONTROL VS Δ INTERVENTION)
**Oxford NOTECHS II**	72.88 (8.65)	72.54 (4.78)	69.81 (7.52)	75.56 (9.33)	0.058
**WHO Time out attempted: n (%)**	23/26 (88%)	23/25 (92%)	18/21 (86%)	21/24 (88%)	1
**WHO Sign out attempted: n (%)**	1/26 (4%)	3/25 (12%)	0/21 (0%)	12/24 (50%)	0.093
**WHO T/O success**	5/26 (19%)	7/25 (28%)	3/21 (14%)	17/24 (71%)	0.032
**Glitch rate/hr**	9.79 (4.12)	13.20 (5.37)	10.48 (2.68)	4.38 (2.50)	<0.001
**Complication rate (%)**	77 (18%)	98 (28%)	14 (9%)	9 (7%)	0.08
**Length of Stay**	5.2 (7.2)	6.0 (7.9)	2.5 (6.3)	1.6 (4.0)	0.095
**Readmission rate (%)**	3 (1%)	7 (2%)	3 (2%)	12 (9%)	0.33

### Summary of all primary outcome measure results

Table 2 gives a summary of the results. The difference in glitch rate (per hour per operation) between the control and active arms was statistically significant (p = <0.001). The difference in Oxford NOTECHS II scores between the control and active arms was of borderline significance (p = 0.058), with the nursing sub-teams the only team showing significant change (p = 0.016). The difference in WHO time-out attempt was not statistically significant (p = 0.322), however the quality of the WHO time-out increased globally in the active arm (p = 0.032). There was significant difference in the completion of the WHO sign-out in the active but not the control group (p<0.001). Complications and LOS both improved in the intervention group but worsened in the control group after the intervention, whilst readmissions went up in both groups. None of the clinical outcome changes achieved statistical significance.

## Discussion

### Summary

The combined intervention we used in this study resulted in improvements in both process and outcome in the intervention group, which were not seen in the control group. The size of the changes, their uniformity and the lack of any change of a similar nature in the control group strongly suggests that we are observing a real effect rather than the effects of bias, secular trends or random variation, although the clinical outcome changes did not reach significance, probably through lack of statistical power. The study was not powered to detect clinical outcome changes, and the fact that the changes in these objective measures, collected by independent observers blinded to the study allocation details, tended to support the findings of the observational work strengthens our confidence in the findings. This global improvement in outcome measures designed to evaluate the human and system aspects of theatre performance suggests that our intervention was effective in improving process, and appears likely to be effective in improving outcomes. Taken together with the results of our linked studies of the effects of each component (teamwork training or “lean” intervention alone), our findings lend support to the S3 study hypothesis that an intervention which targets more than one portion of the three-dimensional model of safety (system, culture and technology) is more likely to make a substantial impression on the workings of the hospital system than a one dimensional intervention. Our study of CRM training alone produced inconsistent results, with improved teamwork but poorer technical performance than the control group (L Morgan, M Hadi, S Pickering, E Robertson, R Dravid, S Menon, T Dale, D Griffin, G Collins, O Rivera, K Catchpole, P McCulloch, The effect of Teamwork training on team performance and clinical outcome in elective Orthopaedic surgery: A controlled interrupted time series study. BMJ Open, in press] whilst a lean only approach using the same training and support approach provided here did not produce any improvements in the outcomes studied ([New, Hadi, Pickering Robertson, Morgan, Griffin, Collins, Rivero, King, Downham, McCulloch, ‘Lean’ participative process improvement: outcomes and obstacles in trauma orthopaedics. BMJ Open, Under Review]

Controlled studies in this field of work are challenging, but their importance is highlighted by recent major pieces of work whose positive results after intervention would have been accepted as evidence of benefit had the control group not also improved[23, 24]. Hospitals are complex, highly dynamic environments in which it is impossible to isolate improvement programmes from the effects of other initiatives and influences. Contemporary control groups are therefore essential, although creating groups which are close enough to the intervention group to be affected by the same external influences, yet isolated from them enough to avoid “contamination” by learning or involvement in the intervention remains a challenge. In this study this was achieved by studying a control group of staff performing a different range of procedures, although in the same Theatre suite and therefore exposed to the same secular trends. The control group in this study experienced important declines in performance in terms of glitch rate and clinical outcomes across the two observation periods. These were temporally associated with identifiable disruptive changes in the operating suite environment (financial pressures, introduction of a new electronic patient record and the sudden death of a key organisational leader in the operating suite) but the intervention group appeared to be protected from the adverse effects.

The positive outcome of this work supports the effectiveness of the style of “lean” intervention used which is based on strong involvement of frontline staff in choosing the focus of interventions and making the change. We believe, based on our previous experiences, that this results in more sustainable gains than if the intervention work was entirely performed by external advisors or consultants. [25]. However this approach also caused difficulties. It required explanation and explicit support from management to overcome staff “learned helplessness” and encourage them to implement their ideas. In other studies, we have also noted that staff may not always choose projects which seem obviously beneficial to safety and reliability, and the tension between providing guidance and avoiding directing the process with resulting loss of engagement is a difficult balance.

The changes in the process measures were greatest on the system measures (glitch). It may be that the teamwork training intervention, as measured by the Oxford NOTECHS II, experienced a ceiling effect, as the pre-intervention rating of both active and control teams’ non-technical skills was already high. However the fact that the team selected improvement projects which were generally appropriate in terms of the overall objective may be partly due to the effects of the teamwork training in focussing attention on patient safety issues in the workplace.

### Limitations and Problems

#### Difficulties encountered

Despite the encouraging change in the outcome measures, the project experienced a number of barriers. One of these was the difficulty in releasing frontline staff to attend training days and to undertake project work. Despite efforts to use convenient times–including evening sessions, anaesthetic audit days (where the operating lists did not commence until lunchtime) and ad-hoc meetings in the theatre coffee rooms, the persistent difficulties in allowing staff any time for improvement work in the various Trusts involved in our programme pointed to a fundamental barrier to improvement in NHS hospitals. Many highly effective industrial organisations and HROs allocate specific staff time for improvement on a regular basis, and the NHS may need to do the same to allow major improvement to occur. The current focus on keeping activity near to the theoretical maximum at all times militates against this, suggesting that the incentives which drive policy at the more senior levels of Trust management need to be re-thought.

#### Limitations

In this study we captured information on the human and system contribution to work process fluctuations in the anticipation that a lean and teamwork training intervention would reduce their frequency. The observers were not blinded to either the study arm or the intervention, and we could not therefore eliminate the possibility of observer bias in the rating of the post-intervention active and control teams, but there are also indicators which suggest this may not have played a major role. The process measures use semi-objective scales which are designed to provide some protection against this bias. Although in this study the findings did favour the intervention, null or unexpected negative results occurred in several of the linked parallel studies. As noted above, the positive trend of the clinical outcome data cannot be attributed to observer bias. Considering generalizability and sustainability, the intervention used appears applicable to most clinical situations in which staff work together in teams to accomplish complex tasks, and the “buy-in” obtained by seeking staff engagement in devising solutions appears likely to help sustainability[26]. Whether the intervention can be scaled up without infeasible expense is an important question which requires further work. It is clear that the amount of external support provided in this study could not be provided at the level of a whole hospital, so experiments are required in methods of scaling up the intervention without losing its effectiveness. The combination of lean process engineering and teamwork training is a rational and relevant intervention for the modern healthcare system, as it addresses both the inter-human relationship and communication problems and the system rationalisation problems which have been identified as important potential causes of error and harm. The linked design of our programme is intended to allow us to carry out an indirect comparison of interventions via meta—analysis, and this should allow us to comment in due course on whether combined interventions do in fact have superior efficacy as opposed to single dimension interventions, once all the studies are complete.

## Conclusions

A combined approach using lean systems improvement processing and crew-resource management training techniques with a strong emphasis on frontline staff engagement and leadership proved successful in improving team process when compared with a contemporary control group, with a trend towards improved outcomes. Difficulties encountered included the tension between staff ownership and control of the direction of the intervention, and the problems of providing staff time for improvement activities in NHS hospitals as currently organised. These results support the hypothesis that combined interventions dealing with both system and culture elements are particularly effective, but comparative studies are required to confirm this.
